# Precision Nutrition and Health Outcomes Across the Lifespan †

**DOI:** 10.3390/nu18203424

**Published:** 2026-10-19

**Authors:** Yang (Scarlet) Shi, Alison G. M. Brown, Shilpy Dixit, Eric J. Shiroma, Nicole Farmer, Qing Lu, Beena Sood, Debbie Vitalis, Maliha Ilias, Marrah Lachowicz-Scroggins, Charlotte A. Pratt

**Affiliations:** National Heart, Lung, and Blood Institute, National Institutes of Health, 6705 Rockledge Drive, Bethesda, MD 20892, USA

**Keywords:** precision nutrition, heart, lung, blood, sleep, chrononutrition, lifespan, data harmonization, implementation, physical activity

## Abstract

Precision nutrition is an emerging paradigm that seeks to tailor dietary recommendations and interventions based on individual and population characteristics. Although advances in multi-omics, wearable sensors, and artificial intelligence/machine learning (AI/ML) have accelerated discovery, translation into practice remains limited by fragmented evidence across life stages and insufficient integration of knowledge regarding systems biology and other influences on health. Nutritional needs, vulnerabilities, intervention opportunities, and response heterogeneity evolve dynamically from preconception and pregnancy through infancy, childhood, adolescence, adulthood, and older age. These life stage-specific processes intersect with (epi)genetic, metabolic and microbiome factors, as well as social, psychological, behavioral, and environmental/geographic influences, contributing to heterogeneity in health outcomes. Addressing this complexity requires not only multimodal predictive models but also frameworks that integrate data standards and harmonization, systems biology, implementation science, and community engagement. To provide a structured approach to this complexity, we developed a seven-pillar Precision Nutrition Framework encompassing prevention for all, data sources, quantitative sciences, qualitative sciences, intervention implementation, communities, and education and workforce training. Using this framework and a lifespan perspective, we examine opportunities for promoting cardiovascular, pulmonary, hematologic, and circadian/sleep health, synthesize evidence on key nutrients, dietary patterns, and emerging biomarkers, and adapt and expand the “precision nutrition plate” for implementation research in clinical and community settings. We highlight critical research gaps, including the need for phenotype-guided trials, standardized data integration, and scalable implementation strategies. By integrating biological, behavioral, and broader environmental dimensions across the life course, precision nutrition can advance more effective, accessible, and sustainable approaches for disease prevention and health promotion.

## Simple Summary

Nutritional needs and vulnerabilities evolve from preconception through older age, necessitating tailored interventions to life stage, biology, and context. The precision nutrition framework emphasizes prevention for all, integrates a variety of data, quantitative and qualitative sciences, community engagement, intervention implementation, and workforce training. Data harmonization, multi-omics, wearables and other tools are essential for scaling precision nutrition. Life stage-specific strategies can optimize heart, lung, blood, and sleep health while addressing other health-related issues and implementation barriers. Research and implementation opportunities exist for precision nutrition research across the lifespan.

## 1. Introduction

Nutrition is a cornerstone of health across the lifespan, influencing development, physiological function, and the risk and management of chronic disease. However, inter-individual variability in dietary responses challenges the effectiveness of traditional “one-size-fits-all” approaches to nutritional guidance. This limitation has driven the emergence of precision nutrition, an evolving field that applies the principles of precision medicine [1,2,3] to dietary science by accounting for biological, behavioral, and environmental heterogeneity in shaping nutrition-related outcomes.

Precision nutrition aims to deliver tailored, highly effective and accessible dietary interventions to specific individuals or populations at the opportune time. Achieving this goal requires integration of multidimensional data, including (epi)genetic, metabolic, microbiome, behavioral, and socioeconomic factors [4,5]. Importantly, these factors do not operate in isolation; rather, they interact dynamically across the life course, shaping both nutritional needs and responses to intervention. Incorporating the broad range of influences on health (e.g., geography/environment, income, education, and food access) into analytic frameworks is therefore essential for understanding and addressing differences in diet-related disease risk.

Recent advances in multi-omics technologies, wearable sensors, and artificial intelligence/machine learning (AI/ML) have enabled increasingly sophisticated biostatistical prediction models that can capture this complexity, integrating dietary and health data to identify phenotypic profiles and inform adaptive, data-driven interventions. However, a critical challenge remains in translating these advances into actionable strategies in clinical and community settings. This gap reflects not only technical barriers, such as data harmonization and integration, but also the need for implementation frameworks that ensure feasibility, accessibility and scalability.

Existing reviews of precision nutrition have largely focused on specific life stages, organ systems, or technological domains, limiting their ability to inform integrated, lifespan-oriented strategies. Yet nutritional exposures and intervention opportunities are inherently dynamic, with critical windows across the lifespan during which targeted interventions may have disproportionate and lasting effects on health trajectories. 

**Figure 1:**
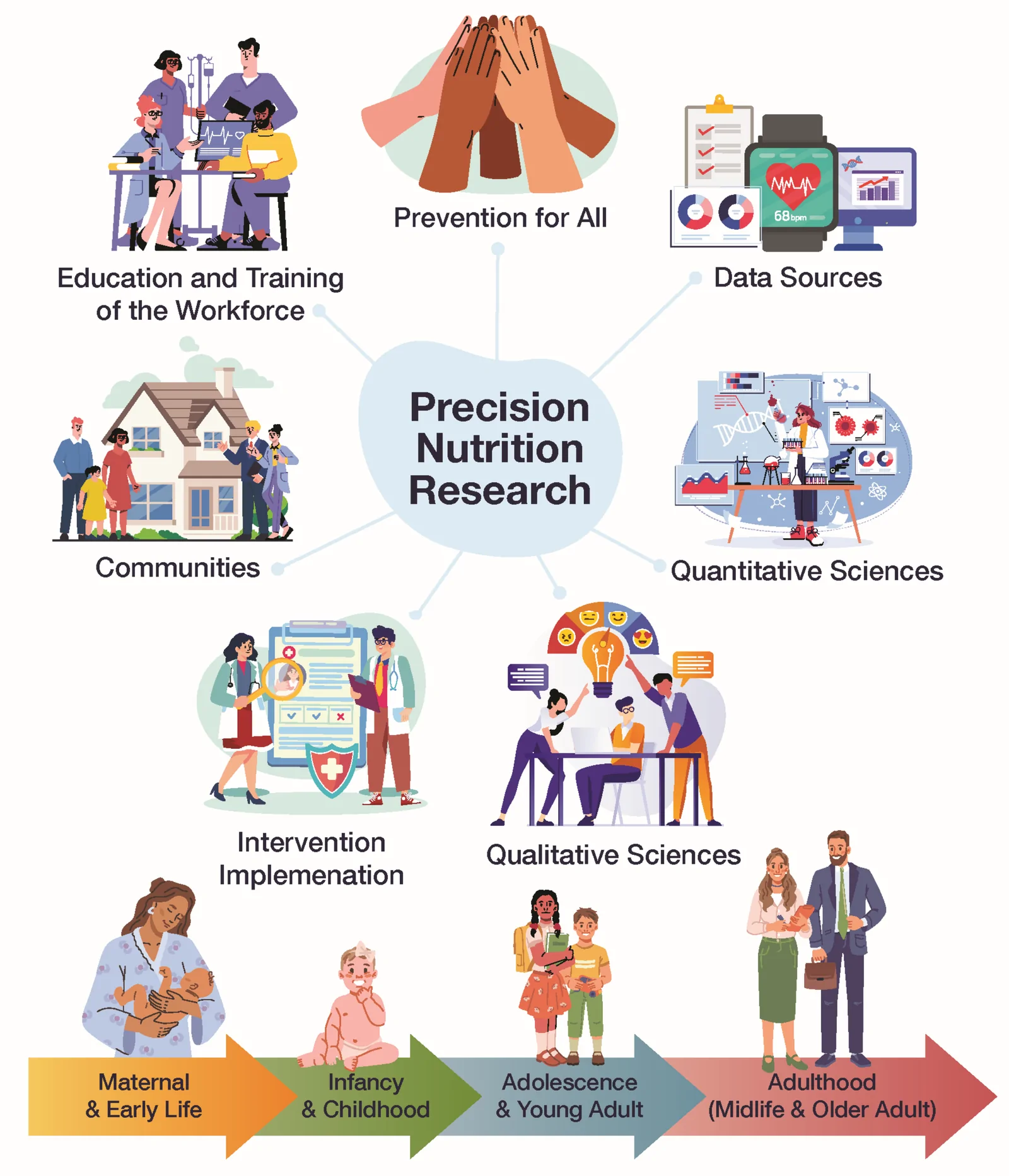
Precision Nutrition Research Framework.

In this paper, we apply a comprehensive, lifespan-oriented precision nutrition framework (Figure 1) to synthesize evidence across cardiovascular, pulmonary, hematologic, and circadian/sleep health domains. We note limited nutrition research in pulmonary, hematologic and circadian/sleep domains. We adapt a seven-pillar precision-prevention model to nutrition science, emphasizing the integration of data harmonization, quantitative and qualitative methods, systems biology, community engagement, and implementation science (Figure 1). We focus on key nutrients, whole foods and dietary patterns that may be at risk of inadequate intake at specific life stages, drawing primarily on literature published since 2020. We selected this timeframe to align with the issuance of the NIH Strategic Plan for Nutrition 2020–2030 [6].

**Figure 2:**
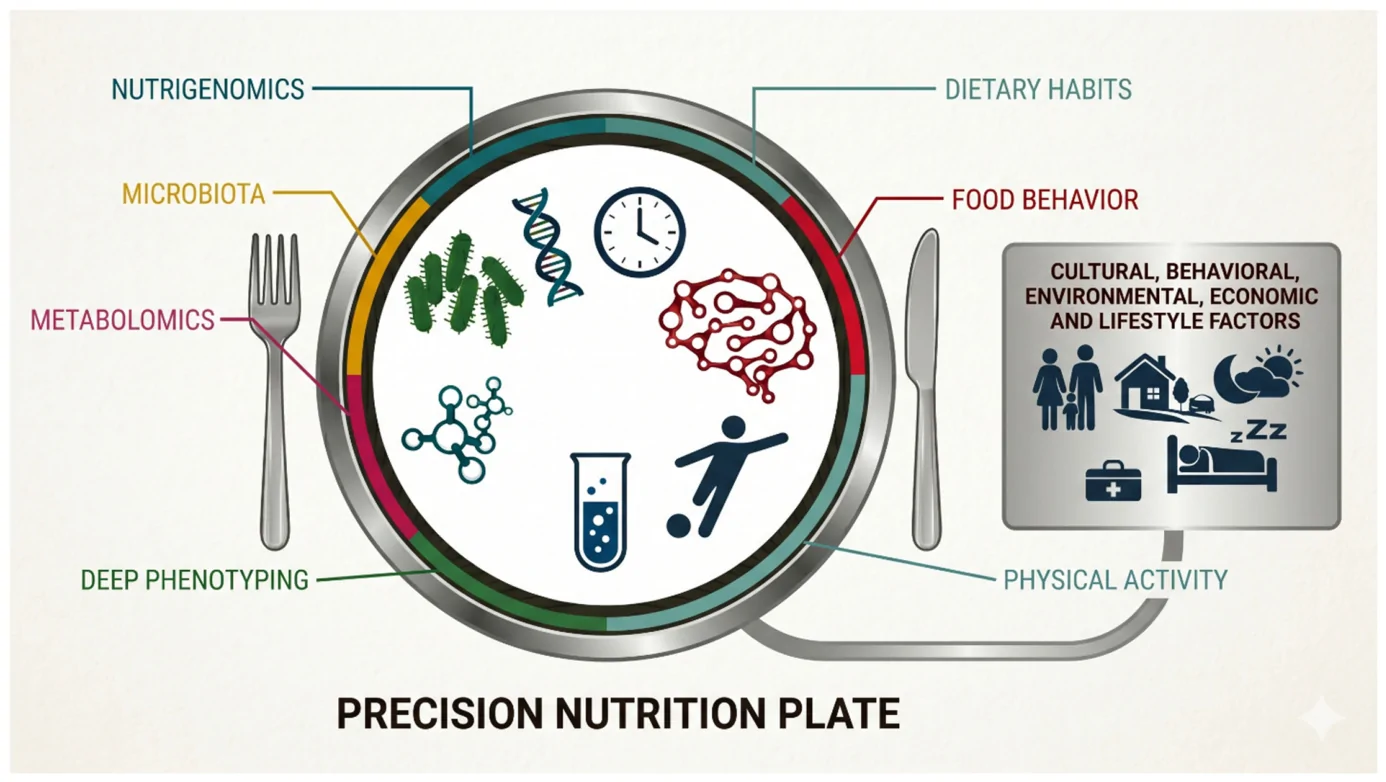
Proposed precision nutrition plate (adapted from De Toro- Martín et al. [7]).

We adapted and expanded the “precision nutrition plate” proposed by de Toro-Martín et al. [7] as a research and practical tool for translating multidimensional evidence into actionable guidance in clinical and community settings (Figure 2). In addition, we identified critical research gaps (Table 1) and opportunities for advancing precision nutrition from a largely conceptual paradigm to an operational, scalable approach for improving health across the lifespan. The identified opportunities align with the NHLBI Strategic Vision [5] and its cross-cutting themes, the NIH Nutrition for Precision Health, powered by the *All of Us* Research Program [8], and the NIH research priorities [9].

## 2. Precision Nutrition Framework

We propose a Precision Nutrition Framework, a conceptual foundation for advancing precision nutrition as an integrated, lifespan-oriented discipline [10]. Rather than considering its constituent domains independently, the framework comprises seven interrelated pillars intended to support the generation, interpretation, translation, and implementation of personalized and population-relevant nutrition strategies. This framework recognizes that precision nutrition is not solely a data-driven enterprise but a systems-level approach that integrates biological and behavioral data with quantitative and qualitative sciences, contextual factors, community engagement, and implementation. This broader perspective is consistent with the NIH Strategic Plan for Nutrition Research, which identifies precision nutrition as a unifying vision and emphasizes individual variability, developing predictive approaches, improving implementation, and building research capacity across the lifespan [5].

## 3. Seven Pillars

The proposed framework comprises seven interrelated pillars: prevention for all, data sources, quantitative sciences, qualitative sciences, intervention implementation, communities, and education and workforce training.

Prevention for all. Precision nutrition begins with a prevention-oriented approach that prioritizes broad population representation while addressing inter-individual variability. Ensuring inclusion of different populations (e.g., across age, sex, familial factors, socioeconomic status, and geographic context) is important for developing and evaluating predictive models and interventions that are accurate, precise, and generalizable. This principle is consistent with published literature positioning precision approaches as complements to, rather than substitutes for, population-level prevention strategies [7,11].

Data sources. Precision nutrition relies on the integration of heterogeneous and complementary data streams. These may include dietary intake assessments (e.g., national surveys such as the U.S. National Health and Nutrition Examination Survey [NHANES]); clinical and physiological measures (e.g., electronic health records, biomarkers, imaging); multi-omics data (e.g., genomics, metabolomics, proteomics, and microbiome profiles); and contextual data related to behavior, lifestyle, environment, and other influences on health. The value of these data lies not only in their individual contributions, but in their combined ability to capture the multidimensional nature of diet–health relationships. Precision-nutrition research increasingly emphasizes multimodal data integration while also recognizing substantial challenges related to measurement, data quality, harmonization, and generalizability [12].

Quantitative sciences. Advanced computational and statistical methods are essential for extracting meaningful insights from complex, high-dimensional data. Technologies such as AI/ML enable the identification of phenotypic subgroups, prediction of individual responses to dietary interventions, and modeling of dynamic interactions among biological, sociodemographic, and environmental factors. These tools support the transition from descriptive associations to quantitative, predictive, and adaptive precision nutrition models. However, their application requires careful attention to data quality, model validation, interpretability, reproducibility, and causal inference [13].

Qualitative sciences. Complementing quantitative approaches, qualitative methods provide critical insights into the social, cultural, and behavioral factors that influence dietary practices. Understanding the “why” and “how” of food choices, including cultural norms, environmental barriers or facilitators to behavior change, and lived experiences, can inform the design of interventions that are feasible, acceptable, and sustainable. Integrating qualitative evidence ensures that precision nutrition remains grounded in real-world contexts in which it is ultimately implemented.

Intervention implementation. The translation of precision nutrition from discoveries into practice requires deliberate focus on intervention design, evaluation, delivery, and implementation. Implementation science frameworks facilitate the development of scalable, adaptable strategies that can be integrated into clinical care and community settings. Accordingly, this pillar extends the framework beyond evidence generation to consideration of how precision nutrition approaches can be implemented at scale. Implementation science is explicitly incorporated into the NIH Strategic Plan for Nutrition Research and has also been identified as an important component of translating precision nutrition into practice [6].

Communities. Community engagement can strengthen the development and implementation of precision nutrition interventions by incorporating local priorities, experiences, cultural practices, resources and constraints. Partnering with communities helps identify contextual factors that affect feasibility, acceptability, uptake and sustainability, and enables the co-design of strategies that improve relevance, trust, and long-term impact. This pillar therefore positions communities not simply as recipients of precision nutrition interventions but as important partners in their development and implementation. Community-engaged approaches have recently been identified as an important opportunity for ensuring that tailored nutrition interventions reach heterogeneous populations [14].

Education and workforce training. Advancing precision nutrition requires a multidisciplinary workforce equipped with research expertise in nutrition science, data science, behavioral science, social sciences, and implementation science. Ideally, training programs would foster interdisciplinary collaboration and develop competencies in emerging methods, including AI/ML, multimodal data integration, and multi-omics approaches, to support innovation and translation. Workforce development is also consistent with the NIH Strategic Plan for Nutrition Research, which identifies training and development of the nutrition scientific workforce as a cross-cutting research area [6].

Together, these seven pillars constitute a proposed systems-level framework linking discovery, individual and contextual variability, and implementation. When applied across the lifespan, this framework is intended to provide a structured approach for addressing heterogeneity in dietary needs and responses, and for developing, evaluating, and implementing generalizable, scalable, and evidence-based nutrition interventions across populations and settings.

This paper applies this Precision Nutrition Framework alongside a lifespan perspective to synthesize evidence spanning cardiovascular, pulmonary, hematologic, and circadian/sleep health. A recurring methodological challenge across these domains is the integration and harmonization of heterogeneous data sources. This challenge is particularly relevant to precision nutrition because multimodal datasets can differ substantially in measurement methods, formats, temporal resolution, scale, and sources of technical and biological variation.

## 4. Data Harmonization for the Nutritional Exposome

The progression of precision nutrition from a theoretical framework to a practical clinical and public health tool depends critically on the ability to integrate and harmonize large, heterogeneous data across biological, behavioral, and environmental domains. As no single cohort captures sufficient heterogeneity in (epi)genetics, metabolic markers, diet, lifestyle, and environmental factors to develop robust, predictive, and generalizable algorithms, advancing precision nutrition requires the integration of data from both existing and future studies. This challenge extends beyond simple data aggregation; it necessitates rigorous, standardized approaches to harmonizing the full “nutritional exposome”, including dietary intake, metabolomic profiles, microbiome composition, and other influences on health.

Much of the existing evidence base resides in legacy cohorts that were not designed using common data elements or current data standards. Leveraging these resources requires sophisticated post hoc harmonization using established guidelines that map variable definitions across studies while preserving validity [15]. These efforts are essential for enabling cross-cohort analyses and for generating robust, generalizable predictive models. Newer studies have begun to include data standards, common study protocols and interoperability requirements prospectively in the study design. The NIH Nutrition for Precision Health (NPH) program, powered by the *All of Us* Research Program, serves as an example of prospective standardization of data collection: participating centers implement a common study protocol, and NPH data are centrally curated and integrated into the *All of Us* Researcher Workbench using metadata and interoperability standards [8].

Three interrelated challenges currently limit the effective harmonization of nutritional data: statistical comparability of dietary intake; technical variability in multi-omics data; and ethical, legal, and privacy constraints for data sharing. First, achieving statistical comparability of dietary intake remains a major obstacle. Dietary data are inherently complex, often characterized by episodic consumption patterns, frequent zero values, and skewed distributions. Traditional two-part models may fail to capture these features adequately, leading to biased estimates of usual intake, particularly at the tails of the distribution, where clinically relevant thresholds (e.g., deficiency or toxicity) often reside. Recent methodological advances, such as Zero-Augmented Birnbaum-Saunders (ZABS) models, offer improved flexibility for modeling asymmetry and heavy-tailed distributions, thereby enhancing the accuracy of intake estimation [16]. Concurrently, harmonizing food terminology across datasets is being addressed through evolving ontologies like FoodOn [17] and the Food-Biomarker Ontology (FOBI) [18], which provide standardized semantic frameworks for food terminology and support integration of dietary-intake data with metabolomics-derived food biomarkers.

Second, technical variability in multi-omics data introduces substantial challenges for cross-study integration. Batch effects, including systematic, non-biological variations arising from different lab protocols, reagents, or equipment and acquisition parameters, can obscure true biological signals and lead to spurious associations if not properly addressed. Effective harmonization therefore requires data-type–specific correction strategies. For RNA-seq count data, count-aware methods such as ComBat-seq use negative binomial regression to model batch effects while retaining integer-valued counts for downstream analyses [19]. In contrast, proteomic and metabolomic platforms introduce data-type- and instrument-specific sources of technical variation, including batch-dependent shifts and signal-intensity drift. Consequently, harmonization requires platform-appropriate quality control, normalization, and batch- or drift-correction procedures rather than a single universal correction strategy [20,21].

Third, ethical, legal, and privacy constraints limit the sharing of sensitive individual-level data, particularly genomic information. These constraints have accelerated the development of privacy-preserving analytical approaches, including Federated Learning (FL), a machine-learning training technique using only local data across multiple devices or sites and then combining the results without sharing the raw data [22]. When coupled with virtual physiological models integrating real-time sensor data, FL enables simulation of metabolic responses to dietary interventions without sharing raw patient data, thereby supporting in silico clinical trials [23]. Such frameworks are particularly relevant for precision nutrition, where integration of high-dimensional, longitudinal data is essential.

## 5. Precision Nutrition: Cardiovascular Health

Cardiovascular health across the lifespan exemplifies the limitations of one-size-fits-all dietary approaches and highlights the need for precision nutrition strategies. Nutrients and dietary patterns influence cardiac morphogenesis, vascular development, myocardial energetics, endothelial function, blood pressure regulation, and autonomic control; yet responses vary widely due to differences in (epi)genetic, metabolic, and microbiome profiles, as well as sex and life stage. Precision nutrition integrates these multidimensional factors to align interventions with those most likely to benefit. A lifespan perspective is essential, as nutritional exposures during key developmental windows shape long-term cardiovascular outcomes and necessitate tailored, phenotype-informed strategies.

Diet also plays an important role in modulating chronic low-grade inflammation, a key pathway in cardiovascular disease. The 2025 American College of Cardiology (ACC) Scientific Statement [24] highlights that dietary patterns influence inflammatory processes, including immune activation, oxidative stress, and endothelial function, contributing to cardiovascular risk beyond traditional lipid measures. Minimally processed whole-food dietary patterns are associated with lower inflammatory burden, whereas diets high in ultra-processed foods are linked to greater inflammation and metabolic risk. Incorporating inflammatory biomarkers such as high-sensitivity C-reactive protein alongside traditional risk factors may further support precision nutrition approaches by identifying individuals with greater inflammatory burden who may benefit from targeted dietary strategies.

### 5.1. Preconception and Early Life

Maternal nutritional and metabolic status before and during pregnancy can influence fetal heart and vessel formation, placental function, and long-term cardiometabolic risk. Early gestation is particularly sensitive, with nutrient-dependent epigenetic processes influencing heart and vessel formation, myocardial growth, and placental function. Maternal factors, including adiposity, metabolic phenotype, and genetic variation, have been associated with altered nutrient utilization and placental transport, contributing to differences in fetal nutrient exposure and supporting the need for individualized nutritional guidance [25,26,27].

*Folate, vitamin B12, and choline.* These nutrients participate in one-carbon metabolism and are essential for DNA methylation and gene regulation during pregnancy and fetal development [28,29]. While epigenetic regulation is central to development, nutrient-specific causal effects on human cardiac morphogenesis remain incompletely established.

Meta-analyses and observational studies generally link maternal periconceptional folic acid supplementation with lower risk of congenital heart defects (CHDs), although estimates and findings vary across studies and CHD subtypes [30,31,32]. A 2024 case–control study reported a U-shaped association between maternal serum folate in early-to-mid pregnancy and offspring CHD risk: both low and high concentrations carried higher odds than intermediate levels, varying by maternal B12 and homocysteine status [33]. Being observational, it does not establish causality or define an optimal dose; replication and intervention studies are needed before findings can inform individualized supplementation recommendations.

Maternal B12 status has also been associated with offspring cardiovascular measures. Lower maternal B12 was linked to reduced heart-rate variability (HRV) in children in a small cohort, although the long-term clinical significance of these findings is uncertain [34]. Maternal vitamin B12, folate, and homocysteine concentrations have additionally been associated with differences in offspring cortisol and cardiovascular responses to psychological stress [35]. Choline also contributes to cardiac gene expression [28,29], although human evidence linking maternal intake to long-term cardiovascular outcomes remains limited [36]. These observational findings suggest potential developmental links between maternal one-carbon metabolism and offspring autonomic and cardiovascular regulation; however, the lack of definitive clinical trial evidence represents an important research gap, and well-designed prospective intervention trials are needed to establish causality and determine whether optimizing maternal one-carbon nutrient status improves offspring cardiovascular outcomes.

Genetic variation may contribute to interindividual differences in choline metabolism and requirements [37]. The *MTHFR C677T* variant likewise may be associated with reduced enzyme activity and may alter folate and homocysteine metabolism [38]. However, current evidence does not support genotype-specific folic acid dosing recommendations [39], highlighting a precision nutrition gap between identifying metabolic heterogeneity and translating it into effective individualized maternal nutrition strategies.

*Long-chain omega-3 fatty acids (docosahexaenoic acid; DHA).* DHA is a structural component of myocardial cell membranes [40] and supports autonomic maturation [41,42]. Randomized trials demonstrate that higher-dose supplementation reduces early preterm birth primarily among women with low baseline DHA status, but with minimal benefit in those already sufficient, highlighting the potential for status-informed precision nutrition [43,44]. Responses are further modified by *FADS1/FADS2* genetic variants affecting endogenous DHA synthesis, suggesting the potential for genotype- and status-informed approaches, although genotype-guided dosing has not been clinically established. While evidence supports DHA’s role in fetal autonomic function (e.g., effects on HRV) [41,42], there is limited evidence from long-term studies connecting prenatal omega-3 supplementation to tangible, clinical cardiovascular outcomes later in life [45,46].

*Other micronutrients, antioxidants, and dietary patterns.* Several additional micronutrients contribute to fetal cardiovascular development. Iodine supports thyroid hormone–dependent myocardial growth and contractility [47], while magnesium regulates vascular tone, placental perfusion, and electrophysiological stability [48]. Vitamin D supports organogenesis, modulates the renin-angiotensin system, and maintains calcium homeostasis. Lower maternal vitamin D status has been associated with an increased prevalence of CHD [49] and structural abnormalities, for example, heart failure or dilated cardiomyopathy in the offspring [50]. However, cohort data provide little evidence for an association with adolescent blood pressure or vascular health [51]. Interventional evidence is mixed, although an international expert panel reached consensus supporting vitamin D supplementation during pregnancy [52].

Antioxidants (e.g., vitamins C and E, selenium, and polyphenols) may mitigate oxidative stress that impairs cardiomyocyte proliferation and vascular development. However, human evidence directly linking maternal antioxidant supplementation to offspring cardiovascular outcomes is limited, with much of the mechanistic support derived from experimental and animal models [53,54].

In contrast, dietary patterns provide more integrative insights: adherence to Mediterranean-style diets (diets rich in fruits, vegetables, whole grains, legumes, nuts, and unsaturated fats) during pregnancy is associated with favorable maternal metabolomic profiles (e.g., improved lipid handling and one-carbon metabolism) and healthier infant cardiometabolic traits such as appropriate birth weight and improved metabolic markers [55,56,57]. These findings suggest potential advantages of dietary pattern–based approaches as a complementary strategy, but comparative trials are needed to determine their superiority over isolated nutrient supplementation.

*Precision nutrition implications.* Collectively, evidence from cohort studies, clinical trials, and mechanistic research indicates that fetal cardiovascular development is shaped by interactions between nutrient availability and maternal biological context. Precision nutrition strategies that incorporate baseline nutrient status, genetic variation, and metabolic phenotype have the potential to optimize maternal–fetal nutrient matching and improve long-term cardiovascular trajectories [58,59,60].

### 5.2. Infancy and Childhood

Infancy and childhood represent continued periods of cardiovascular plasticity, during which nutrients may reinforce, compensate for, or exacerbate in utero influences and thereby shape myocardial growth, vascular structure, blood pressure regulation, and autonomic balance, with effects that may persist in adulthood. Substantial inter-individual variability in nutrient metabolism supports extending precision nutrition approaches beyond pregnancy into early childhood [61].

*One-carbon nutrients.* Adequate intake of folate, vitamin B12, and choline remains essential for cardiovascular growth [29,62,63,64]. Analyses of pediatric nutrition products indicate that, although choline content is standardized in infant formula during early infancy, total choline supply may vary due to additional, non-declared sources, and infants at risk of deficiency (e.g., preterm infants) may require biochemical assessment to ensure adequacy [63]. Vitamin B12 is similarly important, yet suboptimal status is relatively common in infants, even when intake appears sufficient, reflecting variability in absorption and dependence on maternal status. Notably, vitamin B12 is best evaluated using biomarkers of cobalamin status rather than intake alone, supporting biomarker-guided assessment and supplementation when indicated [64]. Observational data link low infant B12 to higher resting heart rate and reduced HRV, suggesting altered autonomic programming [34]. Both deficiency and excess (e.g., high folic-acid exposure) can perturb metabolic and epigenetic regulation, so achieving the correct balance is critical, although evidence concerning excess folic acid includes experimental animal studies and is strongest for prenatal exposure [65,66]. Common genetic variants (e.g., in *MTHFR*) further modify individual requirements, but current guidance does not support genotype-specific folic acid dosing [39]. Consistent with findings from peer-reviewed studies, adequate, while not excessive, one-carbon nutrient intake is achieved through breast milk when maternal status is sufficient, nutritionally fortified infant formula, or targeted supplementation in at-risk populations [63,64].

*Iron and myocardial function.* Iron is required for hemoglobin synthesis and myocardial energy metabolism. Iron deficiency, common in infancy, has direct cardiovascular consequences, including tachycardia, increased cardiac output, and impaired myocardial performance [67,68]—effects that are typically reversible with repletion. Risk is highest among preterm infants, those born to iron-deficient mothers, and infants introduced early to cow’s milk. Exclusively breastfed term infants should receive iron supplementation by 4–6 months of age, with introduction of iron-containing complementary foods around 6 months; preterm infants generally require supplementation earlier. Cow’s milk should not be introduced as the main milk drink before 12 months [68].

*Potassium, magnesium, and blood pressure programming.* Potassium and magnesium play complementary roles in early-life blood pressure regulation by supporting vascular tone, renal sodium handling, and endothelial function. Higher potassium intake in childhood—particularly with moderate sodium intake—is associated with lower blood pressure and a blunted rise during adolescence [69], yet intake often declines below recommended levels as children transition from milk to processed foods [70]. Magnesium, a natural calcium antagonist and enzymatic cofactor, promotes vascular relaxation and endothelial health; deficiency is linked to increased vasoconstriction and elevated blood pressure [71,72]. Responses to these minerals vary by genetic, metabolic, and environmental factors. Controlled feeding studies have identified racial differences in renal potassium handling between Black and White adolescent girls: Black participants had lower urinary potassium excretion regardless of sodium intake and greater potassium retention during a low-sodium diet [73]. Rare variants in magnesium transport genes (*TRPM6*, *SLC12A3*) can markedly impair absorption or increase renal loss [74,75]. Integrating information on biological heterogeneity and other contextual factors may help refine mineral recommendations during critical developmental periods. Further studies are needed to establish evidence-based guidance for the general pediatric population.

*Omega-3 fatty acids and vascular development.* DHA remains important for vascular development after birth. In one randomized trial, DHA-enriched formula was associated with modest long-term reductions in blood pressure in later childhood [76]; however, this effect has not been consistently replicated in longer-term studies [77,78], warranting further investigation. Variability in infant DHA may be driven by maternal diet, breastfeeding, and *FADS1/FADS2* polymorphisms [79,80]; however, further investigation is needed to support baseline- and genotype-informed approaches.

*Other micronutrients.* Zinc is essential for postnatal growth, immune function, and antioxidant defense. Limited and inconsistent evidence links zinc to autonomic and cardiovascular regulation in early life, including altered autonomic patterns after prenatal supplementation, while broader evidence for developmental cardiovascular effects includes substantial experimental data [81,82]. Vitamin D affects vascular tone, inflammation, and myocardial function through multiple pathways [83]. Although infant vitamin D supplementation is standard for bone health, evidence that it prevents pediatric hypertension is inconsistent, and randomized trials targeting vascular outcomes could provide helpful information [84].

Evidence from longitudinal studies suggests that healthier dietary patterns established during infancy and childhood, and often maintained into adolescence, are associated with more favorable cardiometabolic risk profiles later in life [85,86,87]. In the Framingham Children’s Study, higher fruit, vegetable, and dairy intake at ages 3–6 years was associated with smaller increases in systolic blood pressure over 8 years of follow-up and lower systolic blood pressure by early adolescence [88]. Similarly, the Cardiovascular Risk in Young Finns Study demonstrated that healthier dietary patterns during childhood and adolescence were associated with more favorable adult lipid profiles, lower C-reactive protein, lower systolic blood pressure, and lower insulin levels, while a traditional dietary pattern high in rye, potatoes, butter, sausages, milk, and coffee was associated with poorer cardiometabolic risk factors [89]. Higher vegetable intake during childhood and adolescence was also inversely associated with adult metabolic syndrome after 21 years of follow-up [89]. More recently, the Avon Longitudinal Study of Parents and Children (ALSPAC) cohort of 14,646 participants, followed from childhood into early adulthood, found that greater adherence to a DASH-style dietary pattern during childhood (ages 7 and 10) was associated with lower cardiometabolic risk scores in adolescence and early adulthood, although corresponding associations were not observed for DASH adherence at age 13 [90].

Collectively, these findings suggest that dietary patterns established early in life and sustained into adolescence may contribute to healthier cardiometabolic trajectories. However, the available evidence is largely observational and remains insufficient to establish a direct causal relationship between childhood dietary patterns and adult cardiovascular disease events.

*Precision nutrition implications.* Although mechanistic and observational evidence supports life-stage–specific nutrient effects, randomized trials in pediatric populations with cardiovascular endpoints remain limited. Future studies incorporating biomarker and genetic stratification could help test and define optimal timing, dosing, and target populations.

### 5.3. Adolescence and Young Adulthood

Adolescence and young adulthood represent critical windows for modifying cardiovascular risk, as rapid physiological, hormonal, and behavioral changes influence nutrient requirements, body composition, and metabolic health. Adequate energy and the core nutrients emphasized early in life remain important, notably protein and micronutrients such as vitamin D, calcium, zinc, iron, magnesium, alongside antioxidants and balanced macronutrient intake [91].

*Diet quality.* Despite the importance of meeting these nutritional needs during adolescence and young adulthood, diet quality often declines during this period. Population data show inadequate intake of fruits, vegetables, whole grains, and key micronutrients, coupled with excess sodium, added sugars, and refined grains. The NHANES data and the Dietary Guidelines show a decline in Healthy Eating Index scores from 61 (ages 2–4) to 51 (ages 14–18), with widespread shortfalls in vegetables (especially dark-green, red and orange), fruits, whole grains, legumes, dairy, and protein-rich foods, and excess intake of added sugars, saturated fat, sodium, and refined grains [91].

Meeting food-group recommendations supports adequate intakes of iron, fiber, polyphenols, unsaturated fats, and potassium, and helps prevent conditions such as iron-deficiency anemia. In contrast, excessive sodium increases hypertension risk, while refined grains may contribute to higher energy intake and obesity risk [91]. Whole-food, dietary pattern-based approaches remain foundational. Precision nutrition may further enhance implementation of cardiovascular prevention strategies, with emphasis on nutrient density and overall diet quality [92].

*Diet-related cardiovascular risk factors.* Obesity is one of the most prevalent diet-related chronic diseases across the life course. Within the U.S., approximately 13–15% of children aged 2–5 years have obesity [93] and about 23% of adolescents aged 12–19 years are affected [94]. In adulthood, the burden of obesity is substantial; during August 2021–August 2023, ~40% (~138 million) of U.S. adults aged 20 years and older had obesity [95]. Other important diet-related cardiometabolic conditions include hypertension, Type 2 diabetes, and hyperlipidemia. Pediatric primary hypertension, affecting ~2–5% of children [96], often tracks into adulthood and increases with age, with prevalence reaching as high as 45% in U.S. adults [96,97]. In 2023, about 59.5% (~43.7 million) of young adults had at least one of 12 chronic conditions assessed, with obesity, depression, and high cholesterol among the most common [98]. Precision nutrition approaches could help address modifiable diet-related risk factors associated with these conditions [92,99].

*Precision nutrition implications.* These trends underscore the opportunities presented by targeted, life stage-specific approaches to obesity and cardiovascular risk prevention. A precision nutrition framework can support this goal. As described by Yoon et al. [92], such approaches integrate population-level characteristics (e.g., age, sex, behavioral, environmental and cultural factors), individual-level factors (e.g., lifestyle, physical activity, body composition, and metabolic markers), and genetic information to generate tailored recommendations. Extending this framework to younger populations could help address dietary gaps, target limiting nutrients and food groups, and reduce the risk of diet-related diseases across the life course.

### 5.4. Midlife, Menopause, and Older Age

Midlife and older adulthood represent phases in which cumulative nutritional exposures, biological aging, and cardiometabolic risk factors converge and may lead to increasing heterogeneity in cardiovascular outcomes. Precision nutrition approaches in these stages may account for sex-specific transitions, changes in body composition, inflammation, and co-occurring conditions.

*Sex-specific cardiovascular aging.* In women, the menopausal transition constitutes a discrete inflection period for cardiovascular risk [100]. Prior to menopause, women have lower rates of coronary heart disease than age-matched men, partly due to endogenous estrogens that support favorable lipid profiles, endothelial function, and vascular compliance. With menopause, declining estrogen is accompanied by higher LDL cholesterol and triglycerides, qualitative changes in HDL, increased arterial stiffness, inflammation, microbiome changes, and a shift toward visceral adiposity [101,102,103,104,105]. These changes collectively promote a more atherogenic, metabolically adverse phenotype and contribute to the steep rise in cardiovascular risk observed in the subsequent decade.

Genetic variation may further modify nutritional vulnerability during this transition. Common polymorphisms in genes such as *phosphatidylethanolamine N-methyltransferase gene (PEMT) and methylenetetrahydrofolate dehydrogenase 1 (MTHFD1)* can reduce endogenous choline synthesis, increasing dependence on dietary intake [106,107]. Carriers, particularly men and postmenopausal women, who lack estrogen-stimulated PEMT activity, may therefore be more vulnerable to inadequate choline intake and associated organ dysfunction.

The menopausal transition offers a strategic window for targeted intervention [100]. Dietary strategies targeting dominant risk phenotypes—such as elevated apoB-containing lipoproteins, triglyceride-rich dyslipidemia, hypertension, or central adiposity—are likely more effective than generalized dietary approaches. Mediterranean-style dietary patterns have strong support from randomized trials and meta-analyses for primary prevention in older adults, including postmenopausal women, with benefits mediated through improvements in lipids, blood pressure, inflammation, and insulin sensitivity [108,109]. In contrast, the Women’s Health Initiative low-fat intervention did not significantly reduce overall cardiovascular events, and effects varied across baseline risk groups. “Phenotype interventions” are needed to address variability in population responses [110,111].

Specific dietary components may confer additional benefits in selected phenotypes. Long-chain omega-3 fatty acids lower triglycerides and may improve endothelial function in individuals with hypertriglyceridemia [112]. Dietary Approaches to Stop Hypertension (DASH)-style diets are particularly effective for blood pressure–dominant phenotypes [113,114]. Isoflavone-containing foods or supplements may modestly improve endothelial function in postmenopausal women with impaired baseline vascular function [115].

In men, cardiometabolic risk often emerges earlier and progresses more gradually, characterized by higher prevalence of visceral adiposity, hypertension, insulin resistance, and atherogenic dyslipidemia, reflecting differences in sex hormones, body-fat distribution, muscle mass, and metabolic responses to dietary intake [116].

Although foundational dietary principles overlap across sexes, dietary priorities may differ by life stage and dominant risk phenotype, consistent with the broader relevance of sex to precision prevention [116]. For example, lipid- and apoB-focused dietary interventions may be particularly relevant for a newly postmenopausal woman experiencing adverse lipid changes, whereas sodium reduction and weight-centric approaches may be prioritized for a middle-aged man with hypertension and metabolic syndrome.

*Older age.* In older adults, CVD prevention increasingly overlaps with other disease management and preservation of functional capacity, as many have established CVD or multiple cardiometabolic comorbidities. Precision nutrition may therefore need to balance cardiometabolic risk reduction with improvement and/or maintenance of functional capacity, muscle mass, bone health, and overall resilience [117,118,119].

Adequate high-quality protein intake becomes critical to mitigate sarcopenia and frailty in older adults, which are conditions linked to worse cardiovascular outcomes and reduced rehabilitation capacity following cardiac events [119,120]. Due to anabolic resistance, older adults often require higher protein intake per kilogram body weight (e.g., 1.2–1.6 g/kg body weight per day), with even distribution across meals supporting muscle protein synthesis [121].

Micronutrient deficiencies become more common with aging due to reduced intake, impaired absorption, health status and medication interactions. Inadequacies in vitamin B12, vitamin D, magnesium, and potassium can exacerbate vascular dysfunction, hypertension, arrhythmia risk, and metabolic dysregulation [71,83,122,123]. Precision approaches therefore emphasize targeted screening and correction of micronutrient deficiencies rather than indiscriminate supplementation, particularly in older adults with multimorbidity, kidney disease or polypharmacy [124,125].

Dietary patterns such as Mediterranean or DASH-style eating plans remain foundational for supporting cardiovascular health, improving selected inflammatory biomarkers [24]. Greater Mediterranean-diet adherence is also associated with lower risk of functional decline and frailty in older adults [126]. Emphasis on whole, minimally processed plant foods rich in antioxidants may help counter “inflammaging” [24,127], though evidence for supplementation of minerals or vitamins beyond diet remains limited [128].

The gut microbiome is an additional modifier of cardiometabolic health in aging. Reduced microbial diversity is linked to inflammation, frailty, and cardiometabolic risk [129]. Diets high in prebiotic fiber and fermented foods may promote a healthier microbial profile with potential benefits for vascular and immune function, although causal evidence is still emerging.

*Precision nutrition implications.* Across midlife and older age, cardiovascular aging reflects the interplay of sex-specific hormonal changes, cumulative metabolic stress, and individual biological variability. Precision nutrition strategies that stratify individuals by measurable phenotypes (e.g., lipid profiles, blood pressure, inflammatory markers, renal function, and body composition) offer a biologically grounded alternative to one-size-fits-all dietary advice.

Despite strong observational and mechanistic evidence, randomized clinical trials explicitly testing phenotype-guided or omics-informed dietary interventions with cardiovascular endpoints remain limited, particularly in older adults with multimorbidity. Future trials integrating dietary interventions with genomic, metabolomic, and clinical risk stratification could help identify responders, refine recommendations, and translate precision nutrition into routine cardiovascular care. Such efforts could attenuate cardiovascular risk while supporting healthier aging and functional independence.

## 6. Precision Nutrition: Lung Health

Lung health across the lifespan is strongly influenced by nutritional exposures during critical developmental windows, beginning in utero and extending through early childhood and adulthood. Epidemiologic and mechanistic evidence indicates that many chronic respiratory diseases, including asthma and chronic obstructive pulmonary disease (COPD), originate during periods of rapid lung development, when environmental and nutritional factors shape long-term respiratory structure and function. Precision nutrition, which integrates genetic, environmental, and physiological variability, offers a framework to optimize lung development and reduce lifelong disease risk as highlighted in two NHLBI-sponsored workshops in 2024 [130,131].

### 6.1. Pregnancy, Perinatal and Infancy

Natural-history and life-course studies of asthma and COPD suggest that vulnerability to adult lung disease is often shaped during fetal life and early childhood, when lung growth and immune development are highly plastic [132]. In the longitudinal, population-based Tucson Children’s Respiratory Study (TCRS), poor growth and nutritional deficits in utero and in childhood predicted spirometric restriction, and greater respiratory morbidity and mortality in adulthood, supporting early nutrition and developmental programming as key determinants of lifelong lung health [133].

Lung development begins early in gestation (around day 22) and continues through childhood and adolescence; peak lung function is typically reached in early adulthood and is driven by biochemical, endocrine, mechanical and anatomical events [134,135,136,137]. Maternal metabolic and nutritional states, including undernutrition, obesity, and diabetes, can affect fetal lung development and are associated with higher respiratory and allergic risk in offspring, such as asthma and COPD, supporting pregnancy as a priority window for intervention. Key nutrients (e.g., vitamins A, D, and E, omega-3 fatty acids, and antioxidants) are essential for proper lung structure and function.

Precision nutrition strategies could assess maternal genetic variants that affect nutrient metabolism and tailor dietary or supplemental interventions accordingly [138]. Vitamin D receptor (*VDR*) polymorphisms have been associated with asthma and allergy susceptibility in a complex and sex-specific manner [139]. Vitamin D insufficiency during pregnancy is linked to impaired fetal lung development and increased risk of wheeze in offspring [140], with vitamin D supplementation during pregnancy resulting in a significantly reduced risk of asthma/recurrent wheeze in the offspring [141]. However, evidence for *VDR* genotype-guided dosing or timing of vitamin D supplementation to reduce childhood respiratory risk is not yet available and represents a research opportunity (Table 1).

Maternal diet may also interact with child genotype in shaping allergic outcomes; for instance, Filaggrin (*FLG*) loss-of-function mutations in children, combined with poor maternal diet during pregnancy, have been implicated in adverse allergy outcomes, highlighting maternal diet as a modifiable risk factor for offspring allergic disease risk [142].

Despite the importance of nutrition during preconception, pregnancy and postpartum, the diet of most women is suboptimal [143,144]. A consistent daily lifestyle that includes adequate sleep and nutrition is routinely recommended to support maternal health and fetal growth [145], but the timing of maternal dietary practices on lung development remains understudied. In parallel, maternal and infant diets, including breastfeeding, shape the infant gut microbiome and its metabolites, which can influence immune development and inflammatory set-points relevant to allergy and asthma [146,147,148,149,150,151]. Progress will likely depend on future studies linking specific dietary interventions to microbiota-derived immunoregulatory metabolites and downstream respiratory phenotypes.

Postnatally, human milk delivers bioactive, personalized nutrition and is linked to better childhood lung function and lower asthma risk [144,152]. It also acts as “chrononutrition” [153]: hormones (glucocorticoids, melatonin), fat, proteins, amino acids, and endocannabinoids vary diurnally, conveying circadian time cues to infants. Formula and milk supplements therefore may have reduced amounts of these bioactive components and diurnal signals. Expressed mother’s own milk and donor milk may not be circadian matched to the infant; feeding temporally mismatched milk could potentially disrupt the infant’s circadian rhythms and impact infant lung development. Professional guidelines rarely address milk’s circadian properties or recommend circadian matching, and the respiratory consequences remain largely untested, representing a critical research gap requiring experimental and clinical investigation.

Postnatal nutrition is especially pertinent in preterm infants, who are at risk for early nutrition deficits and have higher lifetime respiratory morbidity [154,155,156]. Across measures beyond spirometry, individuals born preterm show persistent differences in lung function (e.g., gas transfer and small-airway measures), supporting the need for early-life strategies that protect lung development [157]. In addition, early growth trajectories, preterm birth, and higher infant weight gain are associated with higher childhood asthma risk, suggesting that “catch-up growth” may not be uniformly beneficial for respiratory outcomes [154,155,156]. Individualized parenteral and enteral nutrition in neonatal intensive care units (NICUs) that meet energy, protein, and micronutrient needs is therefore essential. Precision strategies can include biomarker-guided supplementation (e.g., monitoring vitamin A status and tailoring dosing). Vitamin A supplementation has been shown to reduce bronchopulmonary dysplasia (BPD) risk in preterm infants [158], although other studies reported mixed or limited clinical benefit [159,160]. Feeding practices in preterm infants, such as early human milk fortification with a donor milk-derived fortifier and individualized fortification, support somatic growth and respiratory outcomes more effectively than formula feeding or standard fortification [161,162,163]. Because human milk, donor milk, fortified milk, and formula represent heterogeneous interventions, future research studies should specify fortification strategy, gestational age, comparator and outcomes.

In the neonatal intensive care unit (NICU), continuous delivery of nutrition support is common for premature infants. During critical illness in adults, continuous feeding can potentially blunt the circadian rhythms of gut hormones (ghrelin, insulin) and may contribute to metabolic disturbances, immune dysregulation and feeding intolerance [164,165,166]. Timing nutritional support to preserve circadian rhythms (“chrononutrition”) may improve post-ICU recovery. While current guidelines address nutrient quantity and quality in the NICU, strategies to optimize feeding, timing and mode remain largely unexplored and represent an area for future investigation.

BPD is the most common long-term morbidity of premature birth, with increasing incidence despite advances in perinatal care [167]. BPD survivors face increased risk of respiratory infections, persistent airflow obstruction and early-onset COPD [152,168,169]. Infants with BPD have complex evolving nutritional needs and are at high risk of post-natal growth failure; linear growth predicts better outcomes but is often difficult to achieve. Although research emphasizes BPD prevention and early preterm nutrition, there is limited evidence on nutritional care for infants with established BPD and on how needs change as BPD transitions from acute to chronic disease [168].

As infants transition to complementary foods, personalized nutrition can support lung health by promoting a diverse diet rich in fruits, vegetables, whole grains, and healthy fats that fosters a healthy gut microbiome [170], which in turn influences immune responses and lung health. Introducing allergenic foods according to individual risk assessed by family history may reduce allergic diseases and asthma risk [170,171]; genotype-guided timing for introducing allergenic foods is an emerging research direction. Limiting processed foods and sugar-sweetened beverages further protects against respiratory inflammation.

*Precision nutrition implications.* Throughout childhood, precision nutrition requires longitudinal assessment of intake, nutrient status, and environmental exposure. Diets rich in antioxidants (vitamins C and E), omega-3 fatty acids, and fiber correlate with better lung function and lower asthma risk [172]. Children with genetic variants that reduce antioxidant capacity or alter fatty acid metabolism may benefit from targeted dietary interventions or supplementation. Maintaining healthy body weight through diet and physical activity is also essential as obesity increases the risk of asthma and other respiratory conditions [173].

The supply, quality, and consumption patterns of nutrients have shifted over decades and are shaped by various influences (e.g., environment and lifestyle). Maternal and infant diets are modifiable targets, often at low cost, with the potential for lifelong benefits. Precision nutrition offers a promising framework to optimize lung development, prevent respiratory disease, and promote respiratory health from pregnancy through childhood.

### 6.2. Childhood-Adolescence

Optimal lung health from childhood through adolescence is supported by nutrition that meets growth needs while modulating airway inflammation and immune defenses. Energy and high-quality protein underpin somatic and pulmonary growth, and specific micronutrients play distinct roles: iron (oxygen transport), zinc (innate and adaptive immunity), vitamin A (airway epithelial integrity), calcium and vitamin D (skeletal growth and thoracic development), and long-chain omega-3 polyunsaturated fatty acids (EPA/DHA; precursors of pro-resolving lipid mediators). A dietary pattern emphasizing fruits and vegetables (antioxidants and carotenoids), whole grains and legumes (fiber and micronutrients), dairy or fortified alternatives (calcium, vitamin D), nuts/seeds and fish (healthy fats and protein), and limited added sugars and saturated fat aligns with evidence-based recommendations and supports respiratory resilience [91].

During puberty and transition to young adulthood, rapid linear growth, increases in lean mass, and hormonal changes raise requirements for energy, protein, iron (especially for menstruating adolescents), calcium, and vitamin D to achieve peak bone mass and maintain ventilatory mechanics. Diet quality also influences asthma control and lung function: higher intakes of fiber and plant foods are linked to lower airway inflammation, while omega-3 fatty acids may attenuate type-2 inflammation. Emerging evidence also suggests vitamin D can reduce acute respiratory infection risk that can exacerbate asthma, particularly in those with deficiency [174,175]. Because the adolescent period carries heightened risk for excess weight gain, maintaining a healthy body composition via nutrient-dense dietary patterns and physical activity is important for lowering asthma morbidity.

*Precision nutrition implications.* Novel and evolving biomarkers can help personalize nutrition-lung strategies: Examples include serum 25-hydroxyvitamin D; plasma/skin carotenoids as objective markers of fruit–vegetable intake; fractional exhaled nitric oxide as a measure of type-2 airway inflammation (which can be modulated by antioxidant intake); and metabolomic signatures (e.g., oxylipins, short-chain fatty acids) that reflect dietary patterns and airway inflammatory pathways [176,177]. Integrating these measures with dietary guidance and clinical risk factors could improve risk stratification and enable individualized prevention and treatment across the pediatric–adolescent continuum.

### 6.3. Adulthood and Older Adults

Precision nutrition has the potential to advance lung health promotion, especially among adults and the elderly, who bear the greatest burden of chronic respiratory diseases (e.g., COPD, asthma, and pulmonary fibrosis). This approach utilizes biomarkers, multi-omics, and digital health tools to tailor dietary recommendations and nutritional interventions to an individual’s genetic, metabolic and environmental profile.

Epidemiologic and mechanistic data identify several precision nutritional associations. Higher intake of dietary flavonoids correlated with improved respiratory health, including higher forced expiratory volume in one second (FEV1), slower age-related lung function decline, and lower COPD risk and symptoms, particularly among smokers in the large observational UK Biobank cohort study [178]. Higher plasma levels of omega-3 fatty acids have been associated with improved outcomes and longer transplant-free survival in pulmonary fibrosis [179]. Metabolomic studies have identified low urinary carnitine metabolites in severe asthma and implicated specific plasma sphingolipids in COPD severity and exacerbation risk, pointing to potential therapeutic and nutritional targets [180].

Age-related metabolic changes, comorbidities, and increased vulnerability to malnutrition complicate nutritional assessment in older adults. Notably, individuals may present normal or elevated BMI but suffer from cachexia, frailty, or sarcopenia, which are associated with poor prognosis in COPD [181,182]. Body composition measures, such as fat-free mass index (FFMI) in addition to BMI, and unintentional weight loss, combined with molecular biomarkers (e.g., plasma sphingolipids, omega-3 fatty acid levels), may be associated with more accurate risk stratification and enable targeted nutritional support.

*Precision nutrition implications.* Multi-omics together with genetic and microbiome profiling illuminate how diet interacts with inflammation, immune response, and repair pathways. Dysregulation in specialized pro-resolving mediators (SPM), often attributed to low dietary omega-3 intake, is linked to respiratory disease and may offer a novel therapeutic strategy [183]. Altered gut and lung microbiomes in older adults can modify nutrient metabolism and immune function, reinforcing the need for personalized interventions. AI/ML enhances the ability to process complex clinical, molecular, and behavioral data to identify at-risk individuals, predict responses to interventions, and optimize dietary recommendations. However, translation into practice requires rigorous validation; large cohort studies and randomized, placebo-controlled clinical trials are needed to confirm which biomarkers and multi-omic signatures provide meaningful benefits and to establish evidence-based precision nutrition protocols for adults and older adults. By accounting for heterogeneity in aging physiology, comorbidity, body composition, and molecular phenotype, precision nutrition can identify which dietary patterns, supplements, and interventions are most beneficial for specific subgroups, improving respiratory outcomes, preserving function, and enhancing quality of life.

## 7. Precision Nutrition: Blood Health

Blood disorders, including iron-deficiency anemia, sickle cell disease (SCD), thalassemia, and hereditary hemochromatosis, are uniquely sensitive to nutritional exposures because nutrient metabolism directly influences erythropoiesis, iron homeostasis, inflammation, and oxidative stress [184,185,186]. Therefore, precision nutrition for blood disorders could be framed as a lifespan and genotype-guided approach that evolves with changing physiology and disease biology: prevention of deficiency in childhood, genotype-guided therapy for iron-metabolism disorders, anti-inflammatory and micronutrient optimization in hemoglobinopathies, and absorption-modulation in iron-overload states.

In early life, nutrition is important for hematologic development and long-term disease risk. Adequate iron during infancy and childhood is critical for red blood cell production and neurodevelopment. With aging, altered iron absorption and metabolism increase susceptibility to iron-deficiency anemia, especially when dietary co-factors such as vitamin C are not optimal.

Conversely, age-related metabolic changes such as insulin resistance may be associated with perturbed iron handling, raising the risk of iron overload or hepcidin-mediated functional iron deficiency. Across adulthood and older age, precision nutrition may move beyond simple genotype models to account for chronic inflammation, cardiometabolic comorbidity, and polypharmacy, all of which modify nutrient utilization and hematologic stability [184,187]. In this context, nutrition is not merely supportive care but a modifiable determinant interacting with gene regulation, immune function, and red-cell biology.

In women, iron deficiency may occur largely in relation to menstrual losses and other losses (e.g., childbirth). Across adolescence, pregnancy, postpartum, and menopause, precision nutrition may optimize iron stores before conception, personalize supplementation during pregnancy, correct postpartum losses, and avoid unnecessary iron exposure after menopause while still addressing true deficiency [185].

Genotype-guided precision nutrition can be conceptualized in two domains: (1) dietary influences on monogenic hematologic disorders, and (2) common genetic polymorphisms that modify nutrient metabolism and thereby affect blood-cell function and integrity such as those that impact hepcidin [184]. Monogenic hemoglobinopathies, such as SCD and alpha/beta thalassemia, exemplify conditions where nutritional needs change with age and disease severity. Children and adolescents with SCD often exhibit higher resting energy expenditure, growth impairment, and micronutrient deficiencies, while adults face cumulative inflammatory burden and cardiometabolic comorbidity [184]. Reviews of nutritional status in SCD and thalassemia consistently document suboptimal intake of antioxidants, vitamin D, zinc, and other micronutrients [184]. In a randomized trial of omega-3 fatty acids in SCD, the authors observed reductions in inflammatory markers and vaso-occlusive complications [187], suggesting that targeted supplementation may reduce the symptoms associated with SCD, although more research is needed to verify the results. A systematic review of the literature on nutrition in thalassemia noted that supplementation of nutrients such as vitamin D and zinc may be needed [186] as pathophysiology can modify clinical trajectories [187]. Importantly, nutritional objectives shift across life stages from supporting growth in childhood to controlling inflammation and preserving organ function in adulthood. For example, in adults with heart failure, iron deficiency is a common occurrence, yet there are limited large-scale clinical trials on iron supplementation and morbidity and mortality in heart failure patients [188].

*Precision nutrition implications.* As measurement tools and mechanistic insights advance, precision nutrition has the potential to shift hematologic care from reactive supplementation toward proactive, individualized dietary strategies that may modify disease expression and improve long-term outcomes for people with SCD and blood disorders [189,190,191]. Continued integration of genotypes, biomarkers, life-stage biology, behaviors, and environmental exposures could help translate this promise into clinical practice [186,187].

## 8. Sleep and Circadian Nutrition

Circadian rhythms are endogenous 24 h timing systems that align physiology and behavior, including sleep, hormone secretion, and metabolism, with the day–night cycle. Although the master circadian clock in the suprachiasmatic nucleus is primarily entrained by light, peripheral clocks in nearly all tissues respond to energy availability, macronutrient composition, and the timing of food intake, functioning as an upstream nutrient-sensing network and synchronizing multiple biological systems relevant to heart, lung, blood, and sleep health. Different macronutrients and hormones can influence circadian regulation through various signaling pathways. In experimental studies, carbohydrate- and insulin-related signals have been shown to influence peripheral clocks, while high-fat diets can alter circadian gene expression and protein intake may affect metabolic signaling pathways involved in circadian regulation [192].

Experimental and observational studies show that the circadian system integrates dietary signals with developmental and life-stage demands. Misalignment between endogenous circadian rhythms and eating patterns (e.g., late-night or irregular eating) disrupts hormonal regulation, impairs glucose metabolism, promotes inflammation, and increases risks of obesity and cardiometabolic disease, highlighting that the timing of food intake is nearly as important as the type and quality of food that is consumed for cardiometabolic health [193]. While emerging evidence from meal-timing interventions, including time-restricted eating and intermittent fasting, suggests that circadian-aligned eating can improve metabolic outcomes even without caloric restriction, existing research is largely limited to short-term studies in homogeneous adult populations, with critical gaps remaining across the life course or when a particular biological challenge may be especially responsive to timing-based dietary interventions [194].

Integrating circadian biology into precision nutrition frameworks across the lifespan requires tools that characterize individual circadian phenotypes and timing sensitivities. Multi-omics, continuous glucose monitoring, wearable-derived sleep and activity data, and microbiome profiling can be integrated to map each person’s temporal biology. As an example, emerging evidence indicates that maternal circadian rhythms, meal timing, and metabolic signals during pregnancy might influence placental function and fetal clock development, which may contribute to potential long-term offspring cardiometabolic risk through developmental and epigenetic programming in the offspring. Coupling these approaches with AI/ML may enable adaptive models that predict who will benefit from specific meal-timing strategies, at which life stages, and under what environmental or behavioral conditions [195].

*Precision nutrition implications.* To translate chrononutrition into practice, longitudinal, life-course research and community-based interventions that examine how circadian-aligned nutrition interacts with determinants of health may be useful [195]. Understanding contextual factors (work schedules, food access, cultural eating patterns) could support implementation of timing-based dietary strategies at scale. When grounded in robust measurement and social context, circadian-informed precision nutrition holds promise to deliver the right dietary intervention at the right biological time, improving metabolic, sleep and cardiometabolic outcomes across the lifespan.

## 9. Precision Physical Activity Synergy with Precision Nutrition

Precision physical activity (PA) operates on the biological premise that the “dose” of exercise (modality, intensity, frequency, and timing) may be calibrated to the individual’s genomic, metabolic, behavioral, and life-stage phenotype. This approach moves beyond a simple “move more” paradigm, recognizing that identical exercise stimuli produce heterogeneous physiological and metabolic adaptations driven by an individual’s unique exposome. Landmark findings from the Molecular Transducers of Physical Activity Consortium (MoTrPAC, 2024) mapped the temporal dynamics of responses to physical activity across 19 tissues, revealing that PA regulates many cells in the body with distinct sex-specific differences in adipose lipolysis and mitochondrial signaling pathways [196,197].

Many PA adaptations are mediated by exerkines, signaling molecules released from skeletal muscle, liver, and adipose tissue. Lactate is increasingly viewed as a central lacto-hormone, with autocrine, paracrine, and endocrine-like actions [198,199]. Lactate signaling via GPR81 regulates lipolysis and stimulates brain-derived neurotrophic factor (BDNF), linking metabolic and cognitive health [198]. Translating these findings clinically requires life-course tailoring. In pregnancy, targeted PA prescriptions can optimize the “placental proteome,” enhancing nutrient transporter expression to prevent fetal macrosomia [200]. In childhood, high PA levels can blunt the obesogenic effects of polygenic risk (e.g., *FTO* variants), illustrating a potent gene–environment interaction [201].

Precision physical activity also addresses contemporary clinical needs. For example, GLP-1 receptor agonists may lead to rapid weight loss but risk lean-tissue loss; concurrent resistance training is therefore recommended to prevent sarcopenic obesity and preserve basal metabolic rate [202]. For aging populations and previously sedentary individuals, device-measured VILPA of only 3–4 min per day has been shown to be associated with lower all-cause and cardiovascular mortality, especially among women [203,204]. Timing of activity relative to circadian biology is also associated with improved glucose homeostasis and metabolic outcomes, suggesting alignment of PA timing with an individual’s circadian phenotype [195].

Taken together, heterogeneous molecular and physiological adaptations to PA and their interaction with nutrition underscore the synergy between precision PA and precision nutrition as central components of “precision lifestyle medicine.” Calibrating both exercise and diet to genomic, metabolic and temporal phenotypes promises more effective, personalized prevention and treatment strategies, particularly for overweight and obesity control.

## 10. Implementation Science and Precision Nutrition Plate

Despite proven dietary approaches (e.g., DASH) [195,205] and public health dietary guidelines, adoption of evidence-based nutrition interventions remains limited in clinical and community settings [206]. Implementation science, with its focus on the systematic uptake of research findings and evidence-based practices into routine health care and public health settings, is urgently needed to bridge these gaps to ensure the adaptation, adoption, scalability and sustainability of evidence-based nutrition interventions in the communities that need them most [207].

The adapted precision nutrition plate in Figure 2, with its key domains of nutrigenomics, dietary habits, food behavior, physical activity, deep phenotyping, metabolomics, microbiota, and health factors, can be considered a conceptual map for dissemination and implementation (D&I) research for the uptake and scale-up of nutrition-based interventions. The plate provides a practical structure for surfacing barriers and facilitators for the uptake of nutrition-focused intervention research, identifying appropriate implementation strategies, and evaluating clinical as well as implementation outcomes (acceptability, adoption, feasibility, fidelity, sustainability) across varied settings. For example, precision nutrition highlights that conventional dietary assessment tools often miss the nuance of real-world eating behaviors. For dissemination and implementation (D&I) researchers, this underscores the importance of understanding *local* food environments, cultural food practices, and community-specific eating patterns to improve the uptake of any nutrition intervention.

Combining the Precision Nutrition Plate with established frameworks such as the Consolidated Framework for Implementation Research (CFIR) and Reach, Effectiveness, Adoption, Implementation, and Maintenance (RE-AIM) [208] provides a rigorous conceptual basis for studying how precision nutrition interventions can be translated into routine clinical and community settings. For instance, in community-centered D&I research, precision nutrition interventions can be adapted at the community level by systematically characterizing local health burdens, resources, risk factors, and priorities, which align well with frameworks like EPIS (Exploration, Preparation, Implementation, Sustainment) where community context is rigorously incorporated in the design and conduct of D&I research. Community-engaged D&I researchers can therefore use the plate as a checklist of contextual factors to assess and address when designing, adapting, and scaling nutrition interventions in clinical and community settings.

Community engagement and attention to health factors (e.g., environmental, behavioral, economic) are central to successful uptake of evidence-based nutrition interventions. Food insecurity, cost, access, cultural practices and adherence capacity shape whether tailored recommendations are acceptable, feasible and sustainable in the communities that need them. As Villalobos et al. [209] note, deeper and more meaningful community partnerships are needed to codesign interventions that reflect local realities. As an example, the NIH Community Engagement Alliance (CEAL) [210] provides models of academic–community collaborations that center meaningful community engagement in implementation research that is conducted in the context of each community’s specific social drivers of health such as healthcare access and food security.

In summary, the Precision Nutrition Plate offers a blueprint for multicomponent, multilevel implementation research. For example, effectiveness-implementation hybrid studies allow investigators to evaluate not only whether precision nutrition improves clinical and implementation outcomes but also how, for whom, under what conditions, and at what cost it can be integrated into routine care and real-world settings.

## 11. Examples of Research Opportunities in Precision Nutrition

Based on the identified key research gaps in the sections above, we highlight examples of potential research opportunities in precision nutrition across major domains, as summarized in Table 1. It is notable that many of these research opportunities strongly align with the NIH research priority on precision nutrition [9], and the NHLBI Strategic Vision and Refresh [5] to harness data science and new technologies to drive scientific discovery and precision health, and to advance the science on the importance of lifestyle in chronic disease prevention and treatment. The NIH Common Fund, Nutrition for Precision Health, powered by the *All of Us* research program [8], provides opportunities for further research to predict individual responses to food and dietary patterns.

## 12. Summary and Conclusions

This paper highlights how precision nutrition can address heterogeneity in nutrient metabolism, disease risk, and intervention responsiveness to improve heart, lung, blood, and sleep health outcomes. Precision nutrition offers a transformative, lifespan-oriented framework for optimizing health by integrating biological, behavioral, environmental, and other health determinants to deliver targeted dietary interventions. From preconception and early development through older adulthood, nutritional needs and responses vary substantially, underscoring the limitations of one-size-fits-all dietary guidance. At the same time, critical developmental windows, particularly during pregnancy, infancy, and adolescence, represent high-impact opportunities to shape long-term health trajectories.

Advances in multi-omics, AI/ML, and data harmonization approaches are accelerating the ability to identify phenotypic and genotypic drivers of dietary response, enabling more personalized and predictive strategies.

Importantly, translating precision nutrition into practice requires integration with implementation science, community engagement, and consideration of health determinants. The proposed precision nutrition plate, combined with frameworks like RE-AIM, offers a pathway to scale tailored interventions in real-world settings.

Limitations of this paper include a lack of a systematic review of the scientific literature and a focus on heart, lung, blood and sleep research. Despite substantial progress, key research gaps remain, including the need for longitudinal, heterogeneous cohort studies, standardized data integration methods, and clinical trials evaluating precision nutrition strategies across life stages. Addressing these gaps will be essential to realizing the full potential of precision nutrition as a cornerstone of precision prevention and public health, ultimately enabling delivery of the right dietary intervention for the right individual at the right time across the lifespan.

## Figures and Tables

**Table 1:** Examples of Potential Precision Nutrition Research Opportunities Across the Lifespan.

Domain	Examples of Precision Nutrition Research Opportunities
Cardiovascular Health	Randomized clinical trials testing phenotype-guided or omics-informed dietary interventions with cardiovascular endpoints across the lifespan, particularly in older adults with multimorbidity. Trials incorporating genomic, metabolomic, and clinical risk stratification to identify responders and translate precision nutrition into routine care (e.g., dietary pattern–based interventions accounting for metabolic heterogeneity). Randomized clinical trials integrating dietary patterns, inflammatory biomarkers, and multi-omics approaches to identify personalized nutrition strategies that reduce chronic low-grade inflammation and improve cardiovascular outcomes across the lifespan. Pediatric trials to determine optimal dosing and timing of dietary supplementation and assess cardiovascular outcomes. Integrated approaches combining demographics, social drivers of health, physical activity, body composition, and multi-omics to develop personalized dietary recommendations.
Lung Health	Studies on genotype-guided vitamin D supplementation, antioxidants, and essential fatty acids to reduce pediatric respiratory disease risk. Long-term dietary intervention trials in well-defined populations, particularly older adults; precision nutrition studies using novel biomarkers (metabolomics, proteomics, microbiome). Leveraging AI/ML dietary apps and wearable technologies to improve measurement precision. Use of existing cohorts and biomarkers to accelerate discovery. Studies on maternal diet and feeding patterns and their effects on microbiota, immunoregulation, inflammation, and lung development.
Blood Health	Research that examines the effect of nutritional supplementation in mitigating the complications and symptoms associated with sickle cell disease and other blood disorders. Research on mitigating the prevalence of iron deficiency across the lifespan, particularly in childhood and adolescence, and in adults with heart failure.
Sleep and Circadian Nutrition	Genomic- or biomarker-informed long-term meal-timing interventions (e.g., time-restricted eating, intermittent fasting) examining metabolic outcomes across the lifespan. Consideration of time as a biological variable: research that captures biological, behavioral, and life-course complexity and timing to promote circadian approaches in precision, real-world interventions.
Physical Activity–Nutrition Synergy	Integrated precision lifestyle interventions combining dietary and physical activity strategies; trials examining timing and interaction of physical activity and nutrient intake. Use of wearable technologies and multi-omics to characterize individual responses.
Implementation Science	Research to understand the barriers and facilitators impacting the uptake of evidence-based nutrition interventions across populations with heightened risk of CVD, such as pregnant women and older adults, to inform targeted implementation strategies that enhance the acceptability, feasibility, sustainability, and scale-up of interventions in clinical and community settings. Evaluating the adoption, reach, fidelity, feasibility, cost-effectiveness and sustainment of evidence-based nutrition interventions (e.g., the DASH diet) through community-driven strategies in the context of each community’s unique social and cultural drivers of nutrition.

## Data Availability

No new data were created or analyzed in this study. Data sharing is not applicable to this article.
